# Outdoor Temperature Influences Cold Induced Thermogenesis in Humans

**DOI:** 10.3389/fphys.2018.01184

**Published:** 2018-08-23

**Authors:** Jaël R. Senn, Claudia I. Maushart, Gani Gashi, Regina Michel, Murielle Lalive d’Epinay, Roland Vogt, Anton S. Becker, Julian Müller, Miroslav Baláz, Christian Wolfrum, Irene A. Burger, Matthias J. Betz

**Affiliations:** ^1^Department of Endocrinology, Diabetes & Metabolism, University Hospital of Basel, Basel, Switzerland; ^2^Department of Environmental Sciences, Atmospheric Sciences, Basel, Switzerland; ^3^Department of Health Sciences and Technology, ETH Zürich, Zürich, Switzerland; ^4^Department of Nuclear Medicine, University Hospital Zürich, Zürich, Switzerland; ^5^Institute of Diagnostic and Interventional Radiology, University Hospital Zürich, Zürich, Switzerland

**Keywords:** brown adipose tissue, outdoor temperature, cold exposure, cold induced thermogenesis, thermogenesis, energy expenditure

## Abstract

**Objective:** Energy expenditure (EE) increases in response to cold exposure, which is called cold induced thermogenesis (CIT). Brown adipose tissue (BAT) has been shown to contribute significantly to CIT in human adults. BAT activity and CIT are acutely influenced by ambient temperature. In the present study, we investigated the long-term effect of seasonal temperature variation on human CIT.

**Materials and Methods:** We measured CIT in 56 healthy volunteers by indirect calorimetry. CIT was determined as difference between EE during warm conditions (EE_warm_) and after a defined cold stimulus (EE_cold_). We recorded skin temperatures at eleven anatomically predefined locations, including the supraclavicular region, which is adjacent to the main human BAT depot. We analyzed the relation of EE, CIT and skin temperatures to the daily minimum, maximum and mean outdoor temperature averaged over 7 or 30 days, respectively, prior to the corresponding study visit by linear regression.

**Results:** We observed a significant inverse correlation between outdoor temperatures and EE_cold_ and CIT, respectively, while EE_warm_ was not influenced. The daily maximum temperature averaged over 7 days correlated best with EE_cold_ (R^2^ = 0.123, p = 0.008) and CIT (R^2^ = 0.200, p = 0.0005). The mean skin temperatures before and after cold exposure were not related to outdoor temperatures. However, the difference between supraclavicular and parasternal skin temperature after cold exposure was inversely related to the average maximum temperature during the preceding 7 days (R^2^ = 0.07575, p = 0.0221).

**Conclusion:** CIT is significantly related to outdoor temperatures indicating dynamic adaption of thermogenesis and BAT activity to environmental stimuli in adult humans.

**Clinical Trial Registration:** www.ClinicalTrials.gov, Identifier NCT02682706.

## Introduction

The core body temperature of humans and other mammals is relatively constant and numerous physiologic mechanisms exist to maintain it. The physiological adaptation of the body to long-term cold exposure is called cold acclimatization and comprises several mechanisms ranging from vasoconstriction to avoid heat loss up to shivering in order to increase heat production maximally. BAT is a thermogenic tissue which can convert chemical energy from triglycerides or glucose directly into heat in a process called non-shivering thermogenesis or “cold induced thermogenesis” (CIT) (Cannon and Nedergaard, 2004). BAT contains a large amount of mitochondria and is characterized by a unique protein within the inner mitochondrial membrane called UCP1. UCP1 can dissipate the proton gradient, which is built-up across the inner mitochondrial membrane by the respiratory chain (Fedorenko et al., 2012) which represents the molecular mechanism underlying BAT thermogenesis.

The recent rediscovery of active BAT in human adults has renewed the interest in BAT and human thermogenesis (Cypess et al., 2009; van Marken Lichtenbelt et al., 2009; Virtanen et al., 2009).

In addition to the acute activation of thermogenesis in BAT, repetitive cold stimuli lead to a transformation of white adipocytes into brown adipocytes in rodents (Smith and Roberts, 1964) and humans (Yoneshiro et al., 2013). This process has been called “recruitment” of BAT or “beiging” of WAT depots (Lidell et al., 2014). Recently, it has been demonstrated in mice that the conversion of white to beige adipocytes is bi-directional, i.e., can be reversed by several weeks of high temperatures (Rosenwald et al., 2013). In line with this finding, a higher prevalence of brown adipocytes in the retroperitoneal fat compartment of human patients undergoing adrenal surgery had been detected during the cold season as compared to the warm season (Betz et al., 2013). However, the influence of seasonal temperature changes on human EE and CIT is currently not established.

Cold induced thermogenesis can be measured as the difference of whole body EE before and after a cold stimulus. Moreover, BAT activity can be directly visualized by ^18^F-FDG-PET/CT (van der Lans et al., 2014). Several studies have demonstrated that environmental temperature affects BAT activity when assessed by ^18^F-FDG-PET/CT. However, the majority of studies investigating effects of outdoor temperature on BAT are retrospective analyses of routine ^18^F-FDG-PET/CT scans performed throughout the year (Kim et al., 2008; Au-Yong et al., 2009; Cypess et al., 2009; Ouellet et al., 2011). Due to their design, they observed BAT in only a minority of patients and were not able to differentiate whether cool temperatures acutely activated BAT or whether the cold season expanded the thermogenic capacity of the tissue and its prevalence.

Previously, only two studies assessed the effect of seasonal temperature variation on EE and CIT using a prospective design and controlled cold exposure. Both studies found that higher temperatures reduce CIT, but extent of the effect differed markedly (van Ooijen et al., 2004; Yoneshiro et al., 2013). The conflicting results might be due to climatic differences or different ethnic backgrounds.

We, therefore, analyzed the metabolic response to mild cold exposure in 56 healthy volunteers in Basel, Switzerland, which exhibits a temperate oceanic climate, however, with relatively large differences between summer and winter months.

## Subjects and Methods

### Subjects

**FIGURE 1:**
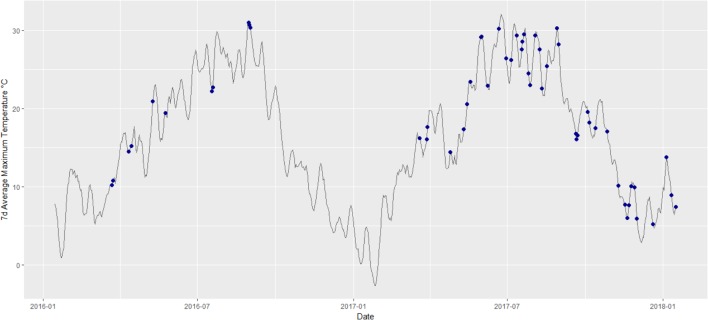
Average of maximum daily temperature and date of the respective study visit (marked by blue dots).

Data were collected from a prospective observational study, MIBAT (clinicaltrials.gov ID NCT02682706), and the screening data from an interventional trial, FluvaBAT (NCT03189511). A total of 56 participants presented to the outpatient endocrine clinic at the University Hospital Basel. At time of inclusion, all participants had been living in Basel and surroundings. EE measurements were performed throughout the year from April 2016 to January 2018 (**Figure 1**). The ethical review board of Northwest and Central Switzerland (EKNZ) approved the studies and all participants provided written informed consent.

### Anthropometric Parameters

In all participants, weight and height was measured. BMI was calculated as body weight in kilograms divided by the square of height in meters [kg/m^2^].

### Energy Expenditure and Cold Induced Thermogenesis

Energy expenditure [kcal/day] was measured during warm conditions (EE_warm_) and after mild cold exposure (EE_cold_) by indirect calorimetry for 30 min using a ventilated hood calorimeter (Cosmed Quark RMR, Cosmed, Rome, Italy). In our laboratory the mean coefficient of variation (CV) for EE_warm_ measured twice in the same subject within 2 weeks was 4.2% (*n* = 17). All measurements took place in an air-conditioned study room at a controlled ambient temperature of 24°C year-round. Subjects fasted during 6 h prior to the study visit to avoid diet-induced thermogenesis. In addition, subjects were requested to refrain from intensive physical exercise for 24 h prior to the respective study visit. For determination of EE_warm_ participants were placed in a hospital bed and were covered with a fleece blanket. After the first measurement the blanket was removed, the subjects were asked to remove all clothes except for t-shirt and shorts. Subsequently, they were exposed to a mild cold stimulus using a water circulated cooling system (Hilotherm Clinic, Hilotherm GmbH, Germany) around the patient’s midsection. The water temperature was continuously reduced by 1°C every 2 min from 25°C to a minimum of 10°C. Participants were periodically asked if they experienced cold or noticed shivering. In case of shivering, they were covered with a blanket for 5 min and the water temperature was raised by 2°C until the shivering stopped. The total cooling time was between 90 and 120 min. During the last 30 min of the cooling the second measurement of EE_cold_ was performed. CIT was defined as the difference between EE_cold_ and EE_warm_. For the statistical analysis, the EE_warm_ and EE_cold_ measurement data of the last 25 min were averaged.

### Laboratory Analysis

In all subjects of the two study groups, serum TSH, fT4, plasma glucose and a lipid profile were performed to ensure euthyroid hormone status and exclude diabetes and lipid disorders. All routine analyses were conducted at the central laboratory of the University Hospital Basel.

As FGF-21 had previously been associated with BAT activity and may be induced by repetitive cold exposure (Hanssen et al., 2015) we measured FGF21 in serum samples collected in separator tubes (Sarstedt, Germany), taken just before the study visits, centrifuged for 10 min at 3000 × *g* and stored at -80°C until analysis. Analysis was performed on the Simple Plex^TM^ Ella microfluidic platform (ProteinSimple, San Jose, CA, United States) using detection antibodies based on the human FGF-21 quantikine ELISA (R&D Systems, Minneapolis, MN, United States). The coefficients of variation for the FGF-21 assay were reported as follows: intra-assay CV 8.2%, inter-assay CV 7.8%.

### Core Body and Skin Temperature

The core body temperature was measured with infrared tympanometry (Braun, ThermoScan PRO 6000, Marlborough, MA, United States) before and after cold exposure.

The skin surface temperature was measured continuously every 60 s during the study visit by wireless iButtons (Maxim Integrated, San Jose, CA, United States) placed at 8 defined body locations. Supraclavicular region (right and left), parasternal at the level of the 2nd intercostal space (right and left), mid-thigh (right and left), middle of the lower arm palmar side and middle of the lower left leg. The temperature data of every location during the last 10 min of warm and cold phase, respectively, were averaged.

The average temperature for the supraclavicular and parasternal region was calculated and designated TempSC for supraclavicular temperature and TempPS for parasternal temperature, respectively. We defined the difference between the TempSC and TempPS as SPTD which has been used as a measure of BAT activity previously (Jang et al., 2014). SPTD was determined for both the warm phase (SPTD_warm_) and for the cold phase (SPTD_cold_).

In order to assess heat loss we calculated the mean skin temperature for warm and cold conditions, respectively, as described previously; Temp_Skin_ = 0.3 × (Temp_Chest_ + Temp_Arm_) + 0.2 × (Temp_Thigh_ + Temp_Leg_) (van Ooijen et al., 2004).

Core body temperature and skin temperatures were compared with the maximum average temperature 7 and 30 days prior to the corresponding study visit, respectively.

### Meteorological Data

Outdoor temperatures were recorded by the Institute for Meteorology, Climatology and Remote Sensing at the University of Basel at an urban meteorological station located close to the University Hospital. Daily mean, maximum and minimum temperatures were averaged over a period of 7 or 30 days prior to the corresponding study visit.

### Statistical Analysis

In order to analyze a possible relation between outdoor temperature and metabolic measurements, we performed linear regression of CIT and EE vs. the average minimum, maximum and mean temperature for the respective study visit. The skin temperature was compared by linear regression only with the average maximum temperature for 7 and 30 days, respectively.

To determine whether age, body weight, height, BMI or sex had an influence on EE_cold_ and CIT in addition to outdoor temperature we first performed linear regression for each parameter. In a second step using multiple linear regression we built a linear model to determine important co-variates. Details on the linear model are provided in the **Supplementary Methods**.

Data analysis was performed using GraphPad Prism Version 7 (GraphPad Software, La Jolla, CA, United States). For multiple linear regression we used R-Software Version 3.4.1 (R-Core-Team, 2017). A *p*-value below 0.05 was considered significant. Data are given as mean ± SD unless stated otherwise.

## Results

### Baseline Data

The anthropometric results are summarized in **Table 1**. The volunteers of the two study populations were comparable in terms of age, BMI and TSH level. In the first study-group (FluvaBAT) only men were included, which were on average significantly taller and heavier than the other group (weight *p* = 0.01, height *p* = 0.0022). Of the 56 participants, 54 (96.7%) were of Caucasian ethnicity and 2 subjects were of a different ethnicity.

**Table 1:** Anthropometric parameters.

	All (*n* = 56)	Group 1 (FluvaBAT, *n* = 31)	Group 2 (MIBAT, *n* = 25)	*p*
Age [years] (range [years])	25.91 5.75 (18-47)	25.52 4.89 (19-36)	26.4 6.73 (18-47)	0.5720
% Female	20	0	44	
Weight [kg]	73.47 9.56	76.37 5.79	69.86 11.96	0.0100^∗^
Height [cm]	178.5 7.43	181.1 6.66	175.2 7.11	0.0022^∗∗^
BMI [kg/m^2^]	22.99 2.15	23.3 1.60	22.61 2.67	0.2351
TSH [mlU/L]	1.87 0.79	1.94 0.80	1.78 0.78	0.5586


*Characteristics of study subjects. The data are given as mean ± SD for all participants and for the respective groups in detail. BMI, body mass index; TSH, thyroid stimulating hormone. ^∗^p < 0.05, ^∗∗^p < 0.01*.

### Metabolic Measurements

The metabolic parameters are described in **Table 2**. EE_warm_ and EE_cold_ were significantly higher in the FluvaBAT study (only male volunteers) than in the MIBAT study (11 female and 14 male volunteers). Importantly, CIT did not differ significantly between the two groups.

**Table 2:** Metabolic parameters.

	All (*n* = 56)	Group 1 (FluvaBAT, *n* = 31)	Group 2 (MIBAT, *n* = 25)	*p*
RQ_warm_	0.77 0.06	0.78 0.06	0.76 0.04	0.1965
EE_warm_ [kcal/d]	1768 278.2	1862 182.9	1650 331.1	0.0036^∗∗^
RQ_cold_	0.77 0.06	0.79 0.07	0.75 0.05	0.0118^∗^
EE_cold_ [kcal/d]	1888 350.1	1979 290.7	1775 388.8	0.0286^∗^
CIT [kcal/d]	120.3 160.7	116.8 147.9	124.7 178.3	0.8560


*Data are expressed as mean ± SD. Asterisks indicate significant differences between the two groups in terms of metabolic measurements. ^∗^p < 0.05, ^∗∗^p < 0.01. Respiratory quotient (RQ): ratio of the amount of carbon dioxide exhaled in a given time to the amount of inhaled oxygen*.

### Energy Expenditure and Outdoor Temperature

**FIGURE 2:**
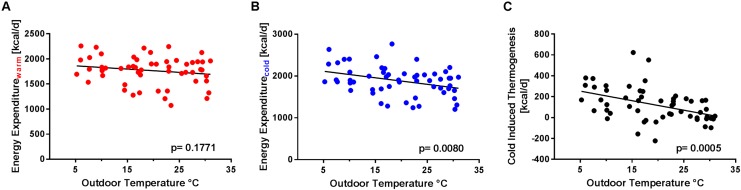
Effect of the average maximum outdoor temperature during the 7 days preceding the study visit on energy expenditure under warm (EE_warm_) and cold (EE_cold_) condition and on cold induced thermogenesis (CIT). **(A)** Outdoor temperature [°C] and EE_warm_ [kcal/d] show no significant correlation. *p* = 0.1771, *R*^2^ = 0.033. **(B)** Inverse correlation between EE_cold_ [kcal/d] and the average maximum temperature during 7 days. **(C)** CIT [kcal/d] (difference of energy expenditure during warm and cold condition) vs. the average maximum temperature [°C] during 7 days. *p* = 0.0005, *R*^2^ = 0.200.

Outdoor temperature did not affect EE during warm conditions (EE_warm_), neither for the 30 days nor for the 7 days (**Figure 2A** and **Table 3**) preceding the study visit.

**Table 3:** Metabolic measurements and outdoor temperature.

	Association with outdoor temperature	Slope ± SE	*y*-Intercept ± SE	*R*^2^	*p*
EE_warm_	Temp7d min	-4.89 6.30	1821 78.56	0.011	0.4404
	Temp7d mean	-6.31 5.47	1862 89.77	0.024	0.2534
	Temp7d max	-6.47 4.73	1894 99.36	0.034	0.1771
	Temp30d min	-0.78 6.73	1776 82.25	0.0003	0.9085
	Temp30d mean	-2.41 5.98	1803 96.14	0.003	0.6884
	Temp30d max	-3.08 5.33	1827 109.8	0.006	0.5660
EE_cold_	Temp7d min	-15.95 7.67	2063 95.64	0.074	0.0422*
	Temp7d mean	-16.58 6.59	2136 108.2	0.105	0.0148*
	Temp7d max	-15.61 5.67	2192 119.1	0.123	0.0080**
	Temp30d min	-10.50 8.36	2002 102	0.028	0.2142
	Temp30d mean	-11.59 7.37	2060 118.5	0.044	0.1214
	Temp30d max	-11.55 6.55	2111 134.7	0.054	0.0837
CIT	Temp7d min	-11.05 3.33	241.7 41.59	0.169	0.0016**
	Temp7d mean	-10.27 2.88	274 47.2	0.191	0.0008***
	Temp7d max	-9.14 2.49	298.5 52.21	0.200	0.0005***
	Temp30d min	-9.72 3.66	226 44.68	0.116	0.0104*
	Temp30d mean	-9.18 3.23	256.3 51.86	0.131	0.0062**
	Temp30d max	-8.46 2.87	283.9 59.04	0.139	0.0047**


*Values are described as mean ± SE. EE_*warm*_, energy expenditure during warm condition; EE_*cold*_, energy expenditure during cold condition; CIT, cold induced thermogenesis. ^∗^p < 0.05, ^∗∗^p < 0.01, ^∗∗∗^p < 0.001.*

Outdoor temperatures significantly correlated with EE_cold_: the largest effect was detected for the average maximum outdoor temperature 7 days prior to the study visit (Temp7dMax, *p* = 0.008, *R*^2^ = 0.123, **Figure 2B** and **Table 3**). For the average outdoor temperature over 30 days, no significant association with EE_cold_ was found.

### CIT and Outdoor Temperature

An inverse relation between CIT and outdoor temperature during both intervals irrespective whether we tested minimum, maximum or mean daily temperatures was found (**Table 3**). The correlation was highest between CIT and the average maximum temperature during 7 days before the visit (*R*^2^ = 0.2000, *p* = 0.0005, **Figure 2C**).

**FIGURE 3:**
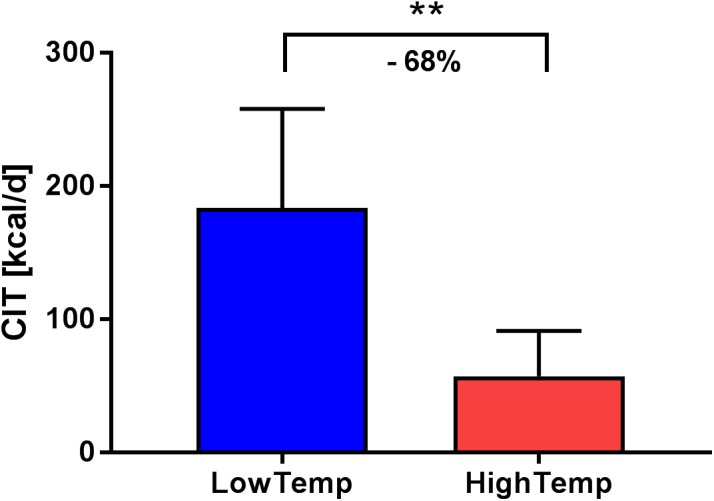
The Difference in CIT compared between HighTemp study visit (average daily maximum temperature during the previous 7 days above the median of 19.5°C) and LowTemp study visit (outdoor temperatures below the median) shows a 68% reduction.

We next compared the CIT values measured at study visits with Temp7dMax temperatures above the median of 19.5°C (HighTemp) to those measured at study visits with outdoor temperatures below the median (LowTemp). Mean CIT at the HighTemp study visits was 57 kcal/d vs. 184 kcal/d for the LowTemp study visits (*p* = 0.0025). Relative CIT (CIT/EE_warm_) was 3.4% at HighTemp study visits vs. 10.0% at LowTemp study visits (**Figure 3** and **Table 3**). Additionally, we calculated the simple linear regression of EE_cold_ and CIT separately for male and female participants. The influence of outdoor temperature was similar in both sexes (**Supplementary Figure S1**).

### Influence of Co-variates on EE_cold_ and CIT

As expected, in simple linear regression EE_cold_ correlated significantly to total body weight (*R*^2^ = 0.419, *p* < 0.0001), height (*R*^2^ = 0.499, *p* < 0.0001), and male sex (*R*^2^ = 0.426, *p* < 0.0001). Age was unrelated (*R*^2^ = 0.008, *p* = 0.518). In multiple linear regression, EE_cold_ was most significantly influenced by outdoor temperature prior to measurement (TempMax7d, *p* < 0.0001) and height (*p* < 0.0001) as well as sex (*p* = 0.005, **Table 4**).

**Table 4:** Multiple linear regression models of energy expenditure during cold conditions and cold induced thermogenesis.

EE_cold_ – full model					

EE_cold_	COLD_EE ∼ TempMax7d + Weight + Height + Sex + Age				

	Coefficients	Estimate	Standard error	*t*-value	*P*
	(Intercept)	-1862.139	906.388	-2.054	0.04518^∗^
	TempMax7d	-16.167	3.449	-4.688	2.16e-05^∗∗∗^
	Weight	5.813	5.247	1.108	0.27319
	Height	20.318	5.850	3.473	0.00107^∗∗^
	Sex (male)	215.156	106.025	2.029	0.04777^∗^
	Age	-6.222	5.266	-1.181	0.24299

*Multiple *R*^*2*^ = 0.7041.*					

**EE_cold_ – reduced model**					

**EE_cold_**	**COLD_EE ∼ TempMax7d + Weight + Height + Sex + Age**				

	(Intercept)	-2367.601	822.364	-2.879	0.00578^∗∗^
	TempMax7d	-16.410	3.426	-4.790	1.43e-05^∗∗∗^
	Height	24.443	4.874	5.015	6.54e-06^∗∗∗^
	Sex (male)	264.818	90.200	2.936	0.00494^∗∗^

*Multiple *R*^2^ = 0.6932.*					

**CIT – full model**					

**CIT**	**CIT ∼ TempMax7d + Weight + Height + Sex + Age**				

	(Intercept)	-1581.082	618.769	-2.555	0.013702^∗^
	TempMax7d	-9.878	2.354	-4.196	0.000111^∗∗∗^
	Weight	-5.304	3.582	-1.481	0.144931
	Height	12.691	3.994	3.178	0.002545^∗∗^
	Sex (male)	-30.836	72.381	-0.426	0.671917
	Age	1.667	3.595	0.464	0.644896

*Multiple *R*^2^ = 0.3457.*					

**CIT – reduced model**					

**CIT**	**CIT ∼ TempMax7d + Weight + Height**				
	(Intercept)	-1347.063	501.537	-2.686	0.00969^∗∗^
	TempMax7d	-9.891	2.319	-4.266	8.44e-05^∗∗∗^
	Weight	-5.398	2.737	-1.972	0.05391
	Height	11.524	3.521	3.272	0.00190^∗∗^


*Multiple *R*^*2*^ = 0.3399. ^∗^*p* < 0.05, ^∗∗^*p* < 0.01, ^∗∗∗^*p* < 0.001.*

Cold induced thermogenesis was not significantly related to sex, age, weight, or BMI in simple linear regression. Height was weakly associated with CIT, (*R*^2^ = 0.08, *p* = 0.035). The best multiple regression model (*R*^2^ = 0.340) for CIT comprised Temp7dMax (*p* < 0.0001), weight (*p* = 0.054) and height (*p* = 0.002) (**Table 4**).

### Relation of Core Body and Skin Temperature to Outdoor Temperature

Body core temperature before and after the cold stimulus, respectively, was not associated with maximum outdoor temperature both for 7 days (warm: *p* = 0.667, cold: *p* = 0.642) and 30 days (warm: *p* = 0.3535, cold: *p* = 0.2532) (**Table 5**).

**Table 5:** Influence of outdoor temperature on core body and skin temperature.

	Association with outdoor temperature	Slope ± SE	*y*-Intercept ± SE	*R*^2^	*p*
Temp7dMax	TempSC_Cold_	-0.01 0.01	35.79 0.16	0.061	0.0665
	Difference between TempSC_Cold_ and TempSC_Warm_	-0.01 0.01	0.05 0.14	0.021	0.2931
	SPTD_warm_	-0.01 0.01	0.80 0.19	0.020	0.2970
	SPTD_cold_	-0.03 0.01	1.57 0.27	0.082	0.0320*
	Mean skin temperature warm	0.01 0.01	33.79 0.22	0.024	0.2562
	Mean skin temperature cold	0.01 0.01	31.71 0.24	0.003	0.674
	Core body temperature warm	-0.002 0.01	36.84 0.10	0.004	0.667
	Core body temperature cold	-0.002 0.004	36.7 0.09	0.005	0.642
Temp30dMax	TempSC_Cold_	-0.02 0.01	35.85 0.17	0.073	0.0434*
	Difference between TempSC_Cold_ and TempSC_Warm_	-0.01 0.01	0.09 0.15	0.028	0.2158
	SPTD_warm_	-0.01 0.01	0.82 0.21	0.019	0.3065
	SPTD_cold_	-0.03 0.01	1.59 0.29	0.071	0.0471*
	Mean skin temperature warm	0.02 0.01	33.7 0.24	0.037	0.15711
	Mean skin temperature cold	0.01 0.01	31.7 0.26	0.003	0.6764
	Core body temperature warm	-0.01 0.01	36.9 0.11	0.016	0.3535
	Core body temperature cold	-0.01 0.01	36.76 0.10	0.027	0.2532


*The outdoor temperature data were compared with the following values: supraclavicular temperature (TempSC) after cold stimulus (TempSC_*cold*_), difference of TempSC before and after cold stimulus, difference between TempSC and TempPS (SPTD, supraclavicular-parasternal temperature difference) during warm (SPTD_*warm*_) and cold condition (SPTD_*cold*_). In addition, mean skin temperature and core body temperature were compared with outdoor temperature. ^∗^p < 0.05.*

While SPTD_warm_ was not correlated to maximum outdoor temperature, SPTD_cold_ correlated significantly, both for 7 days (*p* = 0.032) and 30 days (*p* = 0.0471) prior to the study visit (**Table 5**).

**FIGURE 4:**
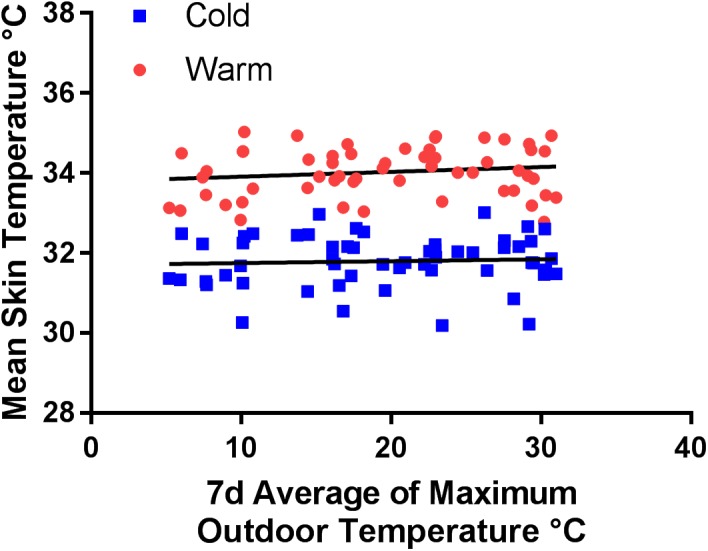
Mean skin temperature [°C] during warm condition (red dots) (*p* = 0.2562, *R*^2^ = 0.024) and in response to the acute cold stimulus (blue squares) (*p* = 0.674, *R*^2^ = 0.003) compared to the average of the daily maximum outdoor temperature over a period of 7 days prior to the corresponding study visit.

The mean skin temperature decreased in response to the acute cold stimulus. However, a connection to the outdoor temperature could not be detected (**Figure 4** and **Table 5**).

### FGF21 and Outdoor Temperature

Mean FGF21 level was 69.04 pg/ml (0.034–386.4 pg/ml). FGF21 correlated neither with any of the baseline characteristics and metabolic measurements, nor with outdoor temperatures (**Table 6**).

**Table 6:** Influence of outdoor temperature on FGF-21 measurements, simple linear regressions.

	Parameter	Slope ± SE	*y*-Intercept ± SE	*R*^2^	*p*
FGF21	CIT	19.19 11.95	38.12 46.09	0.052	0.1151
	AGE	0.78 0.47	23.46 1.81	0.055	0.1036
	Weight	1.12 0.78	69.41 3.00	0.042	0.1568
	Height	0.73 0.57	175.2 2.2	0.034	0.2053
	BMI	0.16 0.18	22.56 0.67	0.018	0.3625
	Temp7dMax	-0.05 1.37	78.38 28.68	0.003	0.7272


## Discussion

In the present study we demonstrated that the seasonal variation of outdoor temperatures significantly influenced the thermogenic response to a mild cold stimulus in healthy human volunteers.

Previously, only two studies had evaluated the impact of seasonal temperature variation on CIT with one study from the Netherlands demonstrating only a mild reduction of CIT of about 40% (van Ooijen et al., 2004) and the other one from Northern Japan showing an almost complete abrogation (-90%) of thermogenic response in summer vs. winter (Yoneshiro et al., 2016). Both studies had performed paired measurements during the warmest and coldest months of the year in the same volunteers. It was, however, not clear whether ethnic or climatic differences led to the disparate results. In our study, measurements were performed throughout the year giving us the opportunity to evaluate a full range of outdoor temperatures. Moreover, while being in the same climate zone as Maastricht, Netherlands, the summer temperatures are generally moderately higher in Basel and the winter temperatures slightly colder and thus closer to the situation in Sapporo. Nevertheless, we observed similar results to the study from Japan with a reduction of CIT during the warmer months vs. cooler months of 68%. This might be an indication that the temperature changes are more important than the ethnic background. However, certain ethnicities have a reduced potential to recruit BAT (Bakker et al., 2014). Our study participants were almost exclusively of Caucasian ethnicity so that we cannot draw any further conclusions on the impact of ethnic background.

Cold induced thermogenesis was not significantly influenced by age, sex, or body weight in our study. Indeed, the results were comparable when we analyzed the CIT and EE_cold_ in both sexes independently.

The missing effect of age and weight is probably due to the relatively young and lean group of healthy volunteers. If we had performed our study also in elderly volunteers we would probably have come to different results with regards to the effect of age as the incidence of cold activated BAT has been shown to be much lower above the age of fifty (Yoneshiro et al., 2011). Interestingly, height was significantly associated with both EE_cold_ and CIT. While EE_cold_ is certainly determined by total body size and as such by height, the effect of height on CIT is not as obvious. A higher stature results in a higher surface-to-volume ratio which leads to increased heat loss in cold environments. We would like to speculate that this might lead to increased CIT but further prospective studies should be performed to investigate this effect.

We did not detect significant associations of the mean skin temperature both before and after cold stimulus and outdoor temperatures. In a previous study, the mean skin temperature at thermoneutrality was slightly, but significantly higher during summer as compared to winter when measured in the same individuals. However, mean skin temperature after mild cold exposure did not differ significantly between summer and winter (van Ooijen et al., 2004). This suggests that the physiological adaption to lower ambient temperatures is mainly induced by increased thermogenesis.

In response to cold exposure or adrenergic stimulation brown adipocytes secrete the peptide hormone FGF21 (Chartoumpekis et al., 2011). Moreover, FGF21 levels were positively associated with cold stimulated BAT activity evaluated by ^18^FDG-PET/CT in humans and serum levels increased after several days of voluntary repetitive cold exposure (Hanssen et al., 2015). We therefore speculated that FGF21 levels might be higher during the cooler season. However, the present results suggest that there is no influence of outdoor temperature on the serum levels of FGF21 and no correlation with CIT. These findings are in line with a small observational study performed in athletes exposed to arctic temperatures during several days (Coker et al., 2017).

Our results have several implications: for research on human thermogenesis and BAT activity the timing of measurements with respect to the season is very important and should be taken into account when designing and evaluating clinical trials, at least in regions with marked seasonal differences in outdoor temperatures. As thermogenesis and active BAT might be useful to increase EE in order to reduce or maintain body weight, individuals might voluntarily seek repetitive mild cold exposure, e.g., by swimming in cool water especially during the warm season. Moreover, from a public health perspective reducing workplace and living room temperatures might be helpful to counteract obesity (Lichtenbelt et al., 2014). In the view of global warming with increasing outdoor temperatures, creating zones of cooler microclimate such as parks in cities could be of great importance for society (Oke, 2017).

Our study has several limitations: First, the exposure of the individual study participant to warm or cool temperatures in the days before the study visit can vary considerably and is difficult to assess systematically. Participants who exhibited significant amounts of CIT during the warm summer months reported of regular camping outdoors or swimming in lakes, however, we did not systematically record data on outdoor activities or clothing habits. Given the effects of ambient temperature on EE seen in this study, future trials should prospectively address this question ideally by recording the ambient and surface temperatures of a subject over longer periods of time with small wearable sensors.

Second, we did not assess BAT activity directly by ^18^F-FDG/PET and can thus not determine the exact contribution of BAT to the effect of outdoor temperatures on CIT. However, reduced amounts of brown adipocytes in the retroperitoneal fat depot during summer months (Betz et al., 2013) and a recent prospective PET/CT study (Bahler et al., 2016) clearly speak in favor of repetitive cold exposure as a significant determinant of BAT activity. Additionally, the difference between supraclavicular and parasternal skin temperature after cold exposure was inversely related to outdoor temperature suggesting increased BAT activity (Jang et al., 2014).

Taken together, we demonstrate a large seasonal variability in CIT in healthy humans probably attributable to BAT. This clearly demonstrates the great physiologic flexibility of the human body in adapting to environmental conditions.

## Author Contributions

MJB designed the study, acquired data for the work, analyzed and interpreted it, drafted the manuscript and revised it critically for important intellectual content. JS and CM acquired data for the work, analyzed and interpreted it, drafted the manuscript and revised it critically for important intellectual content. GG and RV acquired data for the work, analyzed and interpreted it, revised the manuscript critically for important intellectual content. RM, MLd’E, and JM acquired data for the work. AB, IB, MB, and CM contributed to the design of the study, acquired data for the work, analyzed and interpreted it, revised the manuscript critically for important intellectual content. All authors provided approval for publication of the content and agree to be accountable for all aspects of the work in ensuring that questions related to the accuracy or integrity of any part of the work are appropriately investigated and resolved.

## Conflict of Interest Statement

The authors declare that the research was conducted in the absence of any commercial or financial relationships that could be construed as a potential conflict of interest.

## Abbreviations

BAT: brown adipose tissue
BMI: body mass index
CIT: cold induced thermogenesis
EE: energy expenditure
EE_cold_: energy expenditure during cold condition
EE_warm_: energy expenditure during warm condition
FGF 21: fibroblast growth factor 21
fT4: free thyroxine
RQ: respiratory quotient
SPTD: supraclavicular-parasternal temperature difference
SPTD_cold_: supraclavicular-parasternal temperature difference during cold condition
SPTD_warm_: supraclavicular-parasternal temperature difference during warm condition
Temp7d: Temp30d, temperature during 7 days and 30 days, respectively, prior to study visit
TempPS: averaged parasternal skin temperature
TempSC: averaged supraclavicular skin temperature
TSH: thyroidea stimulating hormone
UCP1: uncoupling protein 1
WAT: white adipose tissue
