# Supplementary material for: Outdoor Temperature Influences Cold Induced Thermogenesis in Humans
Source: Front Physiol. 2018 Aug 23;9:1184. doi: 10.3389/fphys.2018.01184 (PMC6115528; doi:10.3389/fphys.2018.01184)
Supplement: Supplementary file 1 [file Table_1.DOCX]

**Supplementary methods:**

**Multiple linear regression**

In order to determine the effect of outdoor temperature together with age, body weight, BMI and sex on EE_cold_ and CIT, we performed multiple linear regression.

We used the *lm* function from the R core *stats* package. CIT and EE_cold_ were inserted as dependent variables. TempMax7d, age, sex and weight and height were used as predictive variables. The quality of the model was assessed by correlating the actual to the fitted values. In a second step, the model was refined to contain only the best predictive variables using the *step* function from the R core *stats* package, which employs the Akaike Information Criterion (AIC) to select the best model. The function removes single variables in a stepwise fashion and compares the resulting model to the previous one using the AIC.

**Supplementary data:**

**Supplementary Figure 1:**

Effect of the average maximum outdoor temperature [°C] during the seven days preceding the study visit on cold induced thermogenesis in male (A) and females participants (B). While the correlation was comparable in both sexes (male: R^2^=0.194, female: R^2^=0.242), the result for the female group failed to reach significance due to the relatively small number of participants (male: p=0.0025, n=45; female: p=0.124, n=11).

Energy expenditure during cold conditions was significantly influenced by outdoor temperature in both sexes; male, R^2^=0.158, p=0.0069 (C); female, R^2^=0.647, p=0.0029 (D).
