# Supplementary figures and images for: Outdoor Temperature Influences Cold Induced Thermogenesis in Humans
Source: Front Physiol. 2018 Aug 23;9:1184. doi: 10.3389/fphys.2018.01184 (PMC6115528; doi:10.3389/fphys.2018.01184)

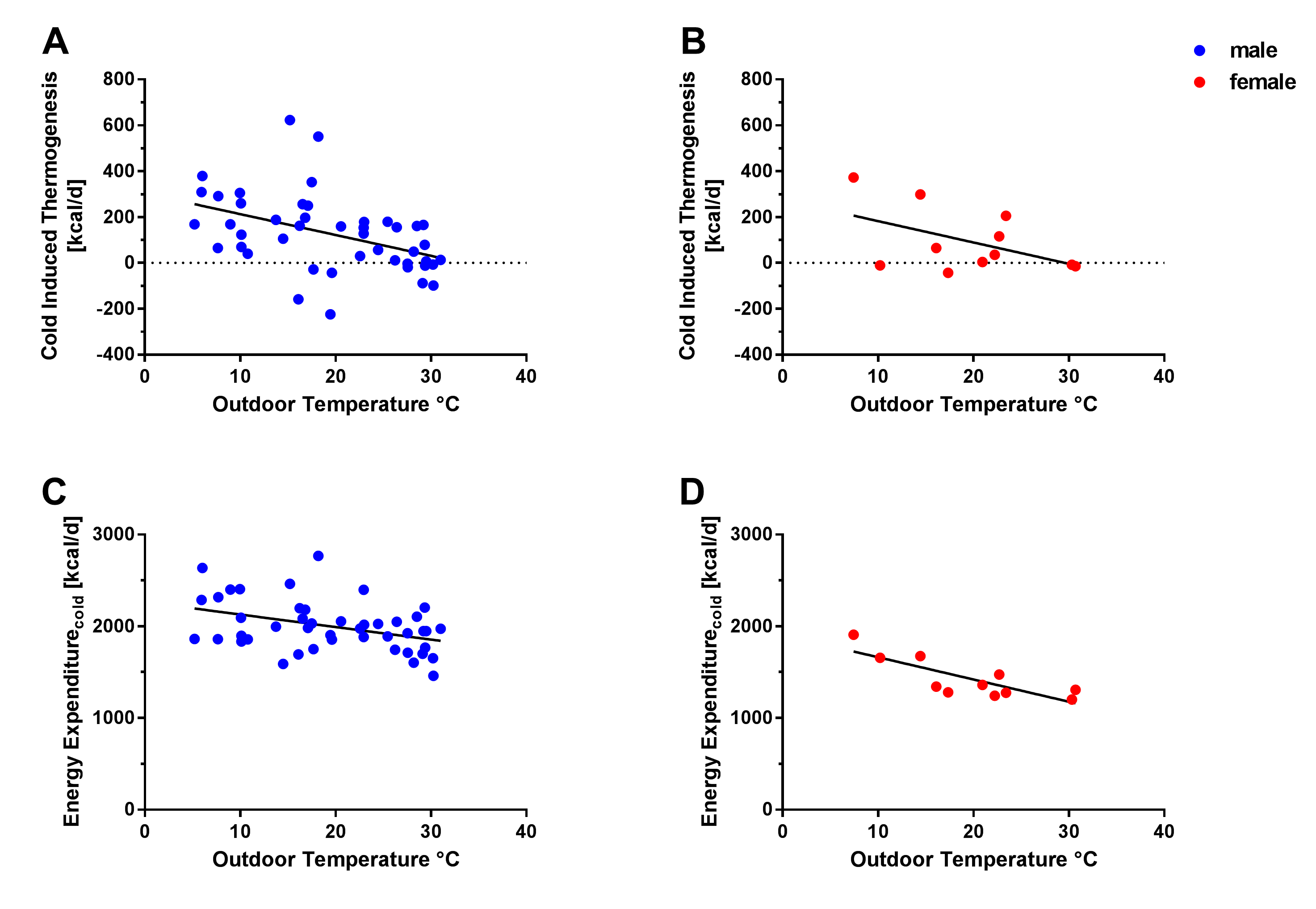

Supplement: Supplementary file 2 [file Image_1.TIF]
